# Antioxidant Valorization of PLE Extracts from Macroalgae (*Cladophora glomerata*): In Vitro Assessment of Nanoemulsions Against Oxidative Stress

**DOI:** 10.3390/antiox13111370

**Published:** 2024-11-08

**Authors:** Lucía Sáez-González, Marcos Carreño-Díaz, Gema Blázquez-Abellán, Manuel J. Santander-Ortega, Rosa M. Martínez-García, Luis A. Martínez, Jose A. Carbajal, Lucía Castro-Vázquez

**Affiliations:** 1 NUTRISAF Research Group, Faculty of Pharmacy, University of Castilla-La Mancha (UCLM), Avda. Doctor Jose María Sanchez Ibañez. S/N c.p., 02008 Albacete, Spain; lucia.saez@uclm.es (L.S.-G.); gemma.blazquez@uclm.es (G.B.-A.); josea.carbajal@uclm.es (J.A.C.); 2 DEVANA Research Group, Pharmaceutical Technology Area, Faculty of Pharmacy, University of Castilla-La Mancha (UCLM), 02008 Albacete, Spain; marcos.carreno@alu.uclm.es (M.C.-D.); manuel.santander@uclm.es (M.J.S.-O.); 3 NUTRISAF Research Group, Departamento de Enfermería, Fisioterapia y Terapia Ocupacional, Facultad de Enfermería, University of Castilla-La Mancha, 16071 Cuenca, Spain; rosamaria.martinez@uclm.es

**Keywords:** *Cladophora glomerata*, plant extracts, bioactive compounds, macroalga, polyphenols, flavonoids, tocochromanols, carotenoids, PLE, antioxidant activity, nanoparticles, macrophage, oxidative stress, ROS

## Abstract

Driven by a growing global interest in natural products, macroalgae have emerged as a prime source for nutraceuticals and pharmaceutical applications. In the current research, the effect of oven-drying (OD) and freeze-drying (FD), as well as the pressurized liquid extraction (PLE) conditions, were investigated in relation to the polyphenols, flavonoids, carotenoids, chlorophylls, and tocochromanols levels in *Cladophora glomerata* extracts. The ethanol–water extracts (1:1) obtained with PLE-150 °C from macroalgae oven-dried (OD)-80 °C displayed the highest total polyphenol content (TPC) and total flavonoid contents (TFCs), reaching 29.62 mg GAE/g and 5.92 mg QE/g. Aqueous extracts using PLE-200 °C were also an excellent environmentally friendly option for TPC and TFC isolation, which were the main factors accounting for the ABTS, DPPH, and FRAP antioxidant activity of the extracts. The remarkable effects of drying conditions positively affect the carotenoids, chlorophyll α-tocopherol, and α-tocotrienol levels from extracts showing 1.3–6 times higher amounts in extracts of *Cladophora glomerata* OD at 80 °C compared with other research approaches. Nanotechnology approaches allowed the formulation of antioxidant-loaded nanoemulsions from *Cladophora glomerata* extracts, providing stability and a great internalization rate that ameliorates to 85% the ROS levels, attenuating the oxidative stress induced by H_2_O_2_ in J774.1 macrophage cell line.

## 1. Introduction

The food and pharmaceutical industries are constantly seeking new natural resources for nutritional and nutraceutical applications. In recent years, marine and freshwater algae have garnered considerable attention due to their wealth of bioactive compounds, particularly antioxidants, which have significant health benefits and industrial applications. They are being studied as a potential source of antioxidant products with commercial interest due to their ability to produce pigments and bioactive compounds, including polyphenols, flavonoids, carotenoids, and omega-3 fatty acids, which are vegan-friendly sources of healthy fats. These compounds possess antioxidant properties and contribute to overall health [1].

*Cladophora* spp. is a macroscopic green algae, widely widespread in both marine and freshwater habitats, growing on submerged rocks and stones or floating freely in the water [2]. Species from the *Cladophora* genus are particularly noteworthy among macroscopic green algae for their high content of bioactive compounds, valorizing this macroalgae as a natural source of valuable bioactive compounds. The principal groups of bioactive molecules present in *Cladophora* species include fatty acids, encompassing both saturated and unsaturated forms; sterols, such as β-sitosterol; carotenoids, including β-carotene; polyphenols, terpenoids, such as trans-phytol; and other bioactive compounds, such as glycosides or vitamins, namely ascorbic acid or vitamin C [3,4]. Many studies of the genus Cladophora have shown that the presence of diverse bioactive compounds renders them a highly valuable raw material for nutritional, industrial, and pharmaceutical applications.

Bioactive compounds of *Cladophora* spp. play a pivotal role in mitigating oxidative stress. Due to their antioxidant properties, these compounds could be exploited for therapeutic applications in oxidative stress-related diseases [5], which are linked to numerous chronic diseases, including cardiovascular diseases, neurodegenerative disorders [6], and metabolic syndrome [7]. The health benefits of *Cladophora* spp. algal compounds and their secondary metabolites extend beyond their antioxidant capacity; they also exhibit anti-inflammatory, antimicrobial, anticancer, and antidiabetic properties, which are essential for maintaining overall health and preventing chronic diseases [8,9,10,11,12]. In addition, previous studies have suggested that *Cladophora glomerata* extract exhibits anti-gastric ulcer, analgesic, and hypotensive activities in various in vitro and in vivo experimental models [13].

Sampling procedures, sample handling, and drying temperatures before extraction can modify the macroalgae bioactive compounds. Drying techniques can considerably affect the extraction efficiency of phenolics and other compounds from macroalgae. For example, the oven-drying of red macroalgae at 40 °C allowed obtaining extracts with higher total phenolic compounds, total flavonoid content, and total anthocyanins, as well as higher antioxidant activity when compared with other drying techniques [14]. Nevertheless, Cruces et al. have reported that the freeze-drying of macroalgae samples has been shown to enhance the phenolic content of extracts when compared with other techniques, such as freezing or air drying [15].

Regarding extraction techniques for bioactive compounds from algae, it should be noted that a variety of extraction methods may be employed. The selection of an appropriate method should be based on the characteristics of the extracted compounds. To date, bioactive compounds have been extracted from algal biomass primarily through conventional solvent extraction, which involves the use of organic solvents such as ether, hexane, toluene, benzene, diethyl ether, dichloromethane, isopropanol, chloroform, acetone, methanol, and ethanol. However, current trends suggest that the use of organic solvents should be minimized.

Today, microwave-assisted extraction (MAE) represents an environmentally benign alternative that reduces both extraction time and solvent consumption. However, high temperature and pressure have been shown to result in the degradation and reduction in the yield of phenolic compounds derived from algae [16]. Supercritical fluid extraction (SFE) uses carbon dioxide as a solvent and is applicable for the extraction of non-polar compounds. This technique has been used to isolate mainly carotenoid and phenolic compounds from *Cladophora glomerata* and *Ulva flexuosa* [17]. Ethanol has been used as a co-solvent to enhance the efficiency of the extraction of algal phenolic compounds [18]. Ultrasound-assisted extraction (UAE) is commonly used to extract phenolic compounds. The efficiency of UAE is contingent upon several factors, including temperature, time, and power of the ultrasonic bath. It has been demonstrated that elevated temperatures can enhance the extraction yield of macroalgal biomolecules [19]. To efficiently extract valuable compounds from plant matrices, advanced techniques such as Pressurized Liquid Extraction (PLE) are also used. PLE, performed using the accelerated solvent extractor (ASE), provides a rapid and efficient method to obtain high yields of bioactive compounds from plant matrices [20]. This method utilizes high pressure and temperature to enhance solvent penetration and solubility of target compounds, making it superior to traditional extraction methods in green, red, and brown algae [21]. The extraction of target compounds and their solubility can be controlled by the applied process parameters of each method. For each of the above extraction methods, different process parameters showed a direct influence on the bioactive compounds extracted from the algae, producing extracts of higher value.

However, the use of bioactive molecules for food and beverage supplementation is most often hampered by stability limitations or inadequate intestinal absorption. Therefore, once extracted, these bioactive compounds need to be effectively delivered to their site of action in biological systems. Here is where nanoemulsions (NEs) can play a key role in enhancing absorption. NEs are excellent carriers for bioactive molecules due to their high surface area and ability to encapsulate hydrophilic and hydrophobic compounds [22]. The small droplet size of NEs increases the stability and bioavailability of bioactive compounds in the organism [4]. Interest in algae NEs has grown exponentially in the last five years [23,24]. NEs may be a promising delivery system for *Cladophora* spp. extracts, which have been investigated very little [10].

In summary, *Cladophora glomerata* algae is one of the best-known botanical sources of antioxidant compounds. However, there are few publications describing the effect of prior drying on the isolation of bioactive compounds.

The aim of this research is to characterize the impact of drying on the levels of bioactive antioxidants, including phenolics, flavonoids, carotenoids, chlorophyll, and tocochromanols from *Cladophora glomerata* green macroalgae. Furthermore, the influence of PLE solvents and temperatures on the extraction efficiency of antioxidant compounds was examined to determine the optimal isolation conditions for obtaining extracts with the highest antioxidant potential.

The current work also focuses on the formulation of NEs using soybean oil and Miglyol © to encapsulate the antioxidant extracts of *Cladophora glomerata*. Furthermore, the biological efficacy of these formulations was tested in vitro using macrophage cell models to investigate the antioxidant impact. This approach aims not only to improve the extraction and delivery of *Cladophora glomerata* algal antioxidants but also to explore the practical implications of using these formulations in biomedical applications. By integrating advanced extraction techniques and nanotechnology, this study contributes to the growing field of biotechnology and its application in health.

## 2. Material and Methods

### 2.1. Material

Macroalgae samples (*Cladophora glomerata*) used in this study were cultivated in a bioreactor from the “Viveros y Repoblaciones La Mancha Industry” (Albacete, Spain) with an environmentally friendly approach for sustainable production of algae-based bioactive compounds. For the cultivation of algae, a high light intensity and nutrient-rich hard water with a pH range of 7 to 10 were required. The freshwater *Cladophora glomerata* was certified as an organic production by the Spanish National Research Council (CSIC) of Madrid.

The algae samples collected from the water were put into plastic containers with water coming from the same habitat and transported to the laboratory. Next, they were rinsed with distilled water. Freshwater samples were divided into 3 portions: (a) two fractions were dried in an oven at 60 °C and 80 °C, respectively, until their water content was less than 5% and constant weight; (b) one macroalgae fraction was freeze-dried. All samples were used to obtain extracts by pressurized liquid extraction (PLE). The extracts obtained by each of the systems were subjected to duplicate analysis.

### 2.2. Heat Treatment: Oven-Drying and Freeze-Drying of Cladophora glomerata

*Cladophora glomerata* macroalgae were cleaned with distilled water in the laboratory. Samples were divided into 3 portions: (a) two fractions that were oven-dried at 60 °C and 80 °C, respectively, until their water content was between 9 and 10%; and (b) one algae fraction that was freeze-dried in a vacuum (2.4 × 10^−2^ mBar) for 24 h, previously frozen at −78 °C for 12 h, with a condenser temperature of −49 °C, as previously described Castro-Vazquez et al., 2021 [25]. The biomass resulting from (a) and (b) for every treatment was extracted by ASE, and the resulting extracts were analyzed.

### 2.3. Cladophora glomerata PLE Extraction

The extraction was carried out by means of an accelerated solvent extractor ASE 200 (Dionex Corp, Sunnyuale, CA, USA). Dried *Cladophora glomerata* samples were ground using a coffee mill.

Two grams of ground powder of *Cladophora glomerata* were placed into ASE-stainless steel extraction cells of 11 mL and were placed into the carrousel. The automatic extraction sequence began with the loading of the cell into the oven. Every cell was filled with different solvent/temperature conditions: acetone, ethanol, ethanol–water (50:50), and water at 50 °C, 100 °C, 150 °C, and 200 °C. Then, two static extraction phases lasting 10 min were carried out under 1500 psi (20 min of total extraction) and were proved with each solvent-temperature condition. Each extract was collected into a glass collection vial. The total solvent volume used was 20 mL. Extractions were performed in duplicate. Between extractions, a rinse of the complete system was performed to avoid any carry-over.

Extracts were evaporated using a rotavapor with a vacuum controller (Heidolph, Schwabach, Germany) until the solvent was removed and 1 mL of *Cladophora* macroalgae extract was obtained. Samples were finally filtered through a Whatman No. 1 filter paper. The extracts obtained were protected from light and stored at −20 °C prior to being used to determine all the parameters to be analyzed.

### 2.4. Determination of Total Phenolic Content

The total phenol content (TPC) of PLE extracts from *Cladophora glomerata* was determined according to the Folin–Ciocalteu procedure [26]. Thus, 1.8 mL of deionized water was added to 0.2 mL of each algae extract. Then, 0.2 mL of Folin–Ciocalteu reagent was added, and the tubes were shaken vigorously. After 3 min, 0.4 mL sodium carbonate solution (35% *w*/*v*) was added together with 1.4 mL of deionized water. Samples were mixed and left in the dark for 1 h. The absorbance was measured at 725 nm using a UV–vis spectrophotometer (Lambda 5, Perkin-Elmer, Seer Green, UK), and the results were expressed in gallic acid equivalents (GAEs) using a standard curve. Extracts were further diluted if the absorbance value was measured above the linear range. Results were expressed as mg GAE·g^−1^ algae respective to dry weight (DW).

### 2.5. Determination of Total Flavonoids Content

The total flavonoid content (TFC) was estimated using the method described by other authors [27,28]. The PLE extracts obtained with four different isolation solvents (0.5 mL of 1 mg/mL) were combined with 1.5 mL of methanol. Subsequently, 0.1 mL of 10% aluminum chloride was added, followed by 0.1 mL of 1 M potassium acetate and 2.8 mL of distilled water. The mixture was incubated at room temperature for 30 min. The absorbance was measured by a spectrophotometer at 420 nm. The results were expressed as milligrams of quercetin equivalents (QEs) per gram of *Cladophora glomerata* (mg QE g^−1^ algae).

### 2.6. Carotenoids and Chlorophyll a and b Determination

The contents of carotenoids and chlorophyll a, b from PLE extracts were investigated using UV–visible light spectrophotometry technique according to Dere et al. [29]. The PLE extracts were redissolved in methanol to reach an absorbance range from 0.2 to 0.8. The absorbance of those solutions was verified at 470, 653, and 666 nm. The contents of pigments, including chlorophylls a, b, and total carotenoids, were calculated according to the next equations reported by other authors [30,31].

Chlorophyll a = 15.65 A_666_ − 7.340 A_653_ (1)

Chlorophyll b = 27.05 A_653_ − 11.21 A_666_ (2)

Carotenoids = (1000 A_470_) − (2.860 Chlorophyll a) − (129.2 Chlorophyll b/245) (3)

The absorbances using wavelengths at 470, 653, and 666 nm were represented as A_470_, A_653_, and A_666_, respectively. The total contents were calculated as μg/g.

### 2.7. Tocopherol and Tocotrienol Determination

Analysis and separation of tocopherols and tocotrienols isomers from *Cladophora glomerata* were performed on high-pressure liquid chromatography (HPLC) using an optical detector (UV–vis).

The PLE extracts, after filtration (0.20 µm, polyester membrane Chromafil PET 20/25), were injected in duplicate on an amino phase Supelcosil LC-NH2-NPHPLC column (250 9 4.6 mm IDx 5 µm particles). The chromatography conditions were the following: an isocratic mobile phase consisting of isopropanol: acetonitrile 70:30 *v*/*v*, a flow of mobile phase of 1 mLmin^−1^, injection volume of 20 µL and a wavelength of 295 nm as a compromise for the simultaneous detection of each compound. Quantification of tocochromanols was made by means of external standard calibration lines. The calibration curves of six points were obtained, covering a concentration range from 0.5 to 100 mg L^−1^. The standards DL-all-rac-a-Tocopherol, DL-all-rac-b-Tocopherol, DLall-rac-d-Tocopherol, and DL-all-rac-c-Tocopherol were supplied by Merk (Darmstadt, Germany).

The tocochromanols identification was confirmed by using an LC-DAD online ESI-MS/MS with a triple quadrupole as a detector in the negative mode [M-H]^−^ according to Castro-Vazquez 2020 [32].

### 2.8. Antioxidant Activity of Algae Extracts

#### 2.8.1. DPPH Radical Scavenging Assay

The DPPH assay was carried out according to the method proposed by Castro-Vazquez et al. [25], where 1,1-diphenyl-2-picrylhydrazyl radical was used as a stable radical. One hundred microliters of different dilutions of extracts were added to 2.9 mL of a 0.06 mM methanol DPPH radical solution. Methanol was used to adjust the zero, and the decrease in absorbance was measured at 515 nm every minute for 25 min in a UV–vis spectrophotometer (Helios, Thermo Spectronic, Cambridge, UK). Only values between 20 and 80% of the initial absorbance of the radical DPPH were taken into consideration. Concentrations were calculated from a calibration curve in the range between 0.1 and 0.8 mM Trolox. Results were expressed in milligrams of Trolox per gram of algae dry weight.

#### 2.8.2. ABTS•+ Radical Scavenging Assay

The method used was the ABTS•+ decolorization assay in accordance with Castro-Vazquez et al. [25] based on the ability of an antioxidant compound to quench the ABTS•+ relative to that of a reference antioxidant such as Trolox. A stock solution of ABTS•+ radical cation was prepared by mixing ABTS solution and potassium persulfate solution at 7 mM and 2.45 mM final concentration, respectively.

The mixture was maintained in the dark at room temperature for 12–16 h before use. The working ABTS•+ solution was produced by dilution in ethanol (1:90 *v*/*v*) of the stock solution to achieve an absorbance value of 0.7 at 734 nm. An aliquot of 20 μL of diluted extract was added to the ABTS•+ working solution. For the blank and standard curve, 20 μL of ethanol or Trolox solution was used, respectively. Absorbance was measured by a UV–vis spectrophotometer at 734 nm immediately after addition and rapid mixing (At = 0) and then every minute for 5 min. Readings at t = 0 min (At = 0) and t = 5 min (At = 5) of the reaction were used to calculate the percentage inhibition value for each extract. A standard reference curve was constructed by plotting the % inhibition value against Trolox concentration (0.1–0.8 mM). The radical scavenging capacity of the extracts was quantified as milligrams of Trolox per gram of algae dry weight.

#### 2.8.3. FRAP Assay

The FRAP assay was performed as previously described by Castro-Vazquez et al. [25]. This spectrophotometric assay measures the ferric-reducing ability of antioxidants. The experiment was conducted at 37 °C and pH 3.6. In the FRAP assay, antioxidants present in the extract reduce Fe (III)-tripyridyltriazine complex to the blue ferrous form, with an absorption maximum of 593 nm. The assay was performed by means of an automated microplate reader (Tecan GENios Pro (Tecan Ltd., Dorset, UK)) with 96-well plates. Reagents included 300 mM acetate buffer pH 3.6, 40 mM hydrochloric acid, 10 mM TPTZ solution, and 20 mM ferric chloride solution. The working FRAP reagent was freshly prepared by mixing acetate buffer, TPTZ solution, and ferric chloride solutions in the ratio of 10:1:1, and the mixture was incubated at 37 °C. Diluted extract (30 μL) and prewarmed FRAP reagent (225 μL) were put into each well. The absorbance at time zero and after 4 min was recorded at 593 nm. The calculated difference in absorbance was proportional to the ferric-reducing/antioxidant power of the extract. For quantification, a calibration curve of Trolox was prepared. The results were expressed as milligrams of Trolox per gram of dried *Cladophora glomerata* algae.

### 2.9. Formulation of Nanoemulsion Systems from Macroalgae Extracts

Nanoemulsions of *Cladophora glomerata* algae were formulated using the solvent displacement technique [33]. This formulation method allows the formation of the *Cladophora* extract containing oily droplets under mild conditions, which preserves the antioxidant properties of the algae extract along the formulation process [25,34,35]. To analyze the effect of the oily core of the nanoemulsion on the antioxidant performance of the nanoemulsion, we selected a non-antioxidant (Miglyol ©) and an antioxidant (soybean) oil for the formulation of the prototypes.

Briefly, 62.5 μL of either Miglyol © or soybean oil were emulsified using lecithin kindly donated by Cargill (Madrid, Spain), 0.25 mL of ethanolic extract of the *Cladophora*, and 4.75 mL of acetone (Sigma, Barcelona, Spain) under magnetic stirring. The aqueous phase was constituted exclusively of 5 mL of MilliQ water. After emulsification, the formulations were evaporated to a final volume of 5 mL at 37 °C. Hydrodynamic mean size and ζ-Potential of the formulations were evaluated by dynamic light scattering using a Malvern ZS90 (Malvern, UK).

### 2.10. In Vitro Studies in Macrophage Cells: Intracellular ROS Levels Measurements

The effect of nanoemulsions on the intracellular ROS levels was studied in J774.1 macrophage cells by the dichlorofluorescein (DCFH-DA) assay. Macrophages at a concentration of 20,000 cells/well were seeded in 96-well plates and incubated at 37 °C for 24 h. Then, the medium was removed, and different aliquots of extract dispersed in the medium (0.78–78 μL/cm^2^) were added to the cells, which were further maintained at 37 °C for 2 h. Then, cells were washed, and 100 μL of DCFH-DA (20 μM) were added and further incubated at 37 °C for 60 min, protected from light. After those incubations, cells were washed, and 100 μL of H_2_O_2_ 123.5 mM were added to each well and incubated for 30 min at 37 °C protected from light. The measurement of the fluorescent oxidized derivative of DCFH-DA was performed in a plate reader (BMG Labtech, Ortenberg, Germany) using emission and excitation wavelengths of 475 and 125 nm, respectively. The treatments H_2_O_2_ and PBS were considered positive and negative controls, respectively. Once the optimal concentration of *Cladophora* extract concentration was selected, the same protocol was performed for the *Cladophora* nanoemulsions.

### 2.11. Statistical Analysis

Analysis of variance and multivariate analysis were performed using a statistical program, SPSS version 21.0 (SPSS Inc., Chicago, IL, USA). Differences between chemical data about phenolic compounds, flavonoids, tocochromanols, pigments, and antioxidant activity of *Cladophora glomerata* algae were established for significance at *p* ≤ 0.05 by the Student–Newman–Keuls test. Pearson correlation was used to compare the correlation between biomolecules and antioxidant activity. All values are provided as the means ± SD of triplicates.

## 3. Results and Discussion

### 3.1. Total Polyphenol Content

The aim of the research was to find out which prior drying treatments are the most effective in enabling the subsequent higher levels of phenolic compounds in *Cladophora glomerata* samples. Furthermore, the objective was to develop effective PLE conditions for the isolation of total polyphenolic compounds (TPCs) from the alga *Cladophora glomerata* using acetone, ethanol, ethanol–water (1:1), and water to cover a wide range of dielectric constants and, therefore, extract bioactive phenolic compounds of varying polarities.

**Figure 1:**
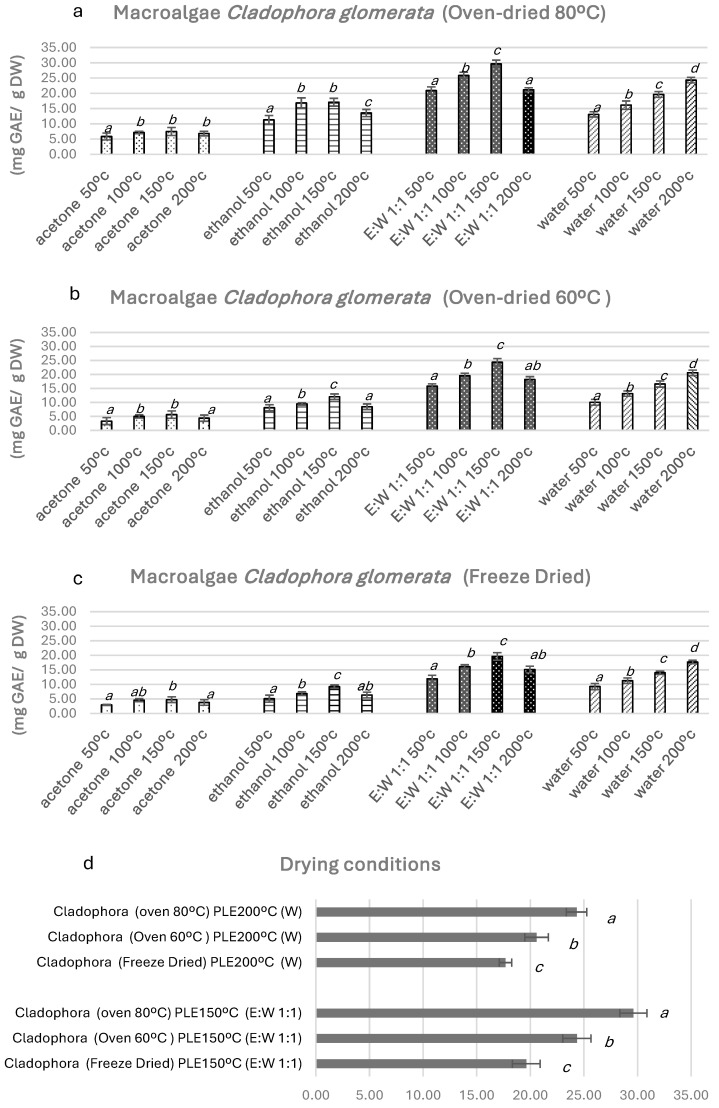
(**a**–**c**) show the total phenolic compounds (TPCs) regarding gallic acid equivalent (GAE) for all the solvents and temperatures tested in *Cladophora glomerata.* (**d**) shows differences between the macroalgae drying method. Different letters in the same column (a–d) denote a significant difference according to the Student–Newman–Keuls test, at *p* ≤ 0.05.

One of the most outstanding results observed was the effect of the previous drying conditions on the *Cladophora glomerata* TPC. Thus, oven-dried (OD) at 80 °C allowed to obtain higher amounts (*p* ≤ 0.05) of extractable phenolic compounds compared to oven-dried (OD) at 60 °C and freeze-dried (FD) (Figure 1) for all extracts, with all solvents and temperatures tested. The results obtained suggest a reasonable release of phenolic compounds as a consequence of the drying temperature, which improves the final TPC levels of *Cladophora glomerata* extracts. Parallel findings reported that this behavior was based on increases in the free fraction of phenolic acids in the ratio of free to glycoside-bound forms in food matrices [25]. This trend has also been described for *Cladophora glomerata* bioactive compounds dried at 105 °C [36].

Regarding PLE solvents, Figure 1 shows that TPCs were extracted more effectively when it was performed using E:W (1:1) > water > ethanol > acetone. It can be concluded that the highest levels of total phenolic compounds were found in PLE extracts with ethanol–water 1:1 (E:W 1:1) in samples previously oven-dried (OD) at 80 °C. This fact indicates that most of the soluble phenolic compounds from this macroalga have a high polarity. Acetone was the least effective extraction solvent, obtaining the lowest TPC values ranging from 2.93 to 7.48 mg g^−1^ d.w. These results are consistent with the polar nature of phenolic compounds and the polarity of the solvents employed.

PLE temperature is also an important parameter to consider when calculating TPC isolation. Therefore, extractions were performed at 50 °C (low temperature), 100 °C, and 150 °C (medium temperature) and 200 °C (high temperature). It can be noted that the lowest extraction efficiency was obtained not only for PLE extracts at 50 °C but also at 200 °C because phenolic compounds can undergo thermal degradation, hydrolysis, and oxidation at high temperatures. Accordingly, 200 °C (using organic solvents) was not a suitable temperature to obtain extracts rich in TPCs, as already noted in other studies [37,38].

The highest content of TPC was found for PLE at 150 °C with ethanol–water (E:W 1:1), reaching 29.62 mg GAE/g d.w. When working at 100 °C, the E:W *Cladophora glomerata* extracts also showed outstanding TPC values, ranging from 16.06 to 25.85 mg GAE/g d.w., but significantly lower (*p* ≤ 0.05) than those obtained at 150 °C. Results are better than those found in the literature for other *Cladophora* species such as *C. surera* from Argentina (2.69 mg GAE/g d.w.) [11], *C. prolifera* and *C. vagabond* from Mexico (1.02–1.95 mg GAE/g d.w.) [39], and *C. ruprestris* and *C. vulgaris* from the Lithuanian coast (1.26–3.52 mg GAE/g d.w.) [40].

The current levels of TPCs in *Cladophora glomerata* samples obtained by PLE at 150 °C with ethanol–water (E:W 1:1) are also more favorable than those obtained using other isolation techniques such as soxhlet or simple maceration (1.32–17.30 mg GAE/g d.w.) [41,42], even higher than those reported by Korzeniowska et al. [43] using UAE and MAE with ethanol and ethanol–water (70–30%), and also higher than 25.1 mg GAE/g d.w. reported by Messyasz et al. [44] using UAE with methanol. However, it should be borne in mind that algal TPC levels can be significantly affected by seasonal variations related to various environmental factors such as light intensity, UV radiation, and nutrient availability; in fact, higher TPC levels generally occur in summer and early autumn, whereas lower levels are observed in winter [45].

The TPC levels obtained through PLE using water as an extractant were particularly noteworthy. The utilization of water in PLE is designated as subcritical water extraction (SWE). The water is heated at temperatures reaching 200 °C, which alters its dielectric constant, rendering it capable of behaving like an organic solvent. To illustrate, the dielectric constant of water at 200 °C is 36, which is nearly equivalent to that of methanol [46].

Our most favorable results were 24.32 mg GAE/g d.w. using PLE-200 °C with water as the green solvent from *Cladophora glomerata* samples previously oven-dried at 80 °C. TPC levels with water were very similar to those obtained with E.W (1:1) at PLE-150 °C and PLE-100 °C: 29.62 and 25.85 mg GAE/g d.w., respectively (Figure 1). Thus, comparing the extraction conditions and their effectiveness, results for PLE with water at 200 °C can be considered a very good alternative for the isolation of antioxidants TPCs instead of organic solvents. In comparison with the existing literature, the present results are 1.5–3 times higher than those previously reported using boiling deionized water, maceration, or water–solvent extraction [47,48,49] and even 5–6 times higher than recent research using ultrasound-assisted extraction (UAE) or microwave-assisted extraction (MAE) with water in *Cladophora glomerata* [43]. This fact supports that the PLE conditions with water at 200 °C proposed here are an excellent environmentally friendly option to perform TPC extraction and highly effective in obtaining *Cladophora* extracts enriched in TPCs.

### 3.2. Total Flavonoid Content

**Figure 2:**
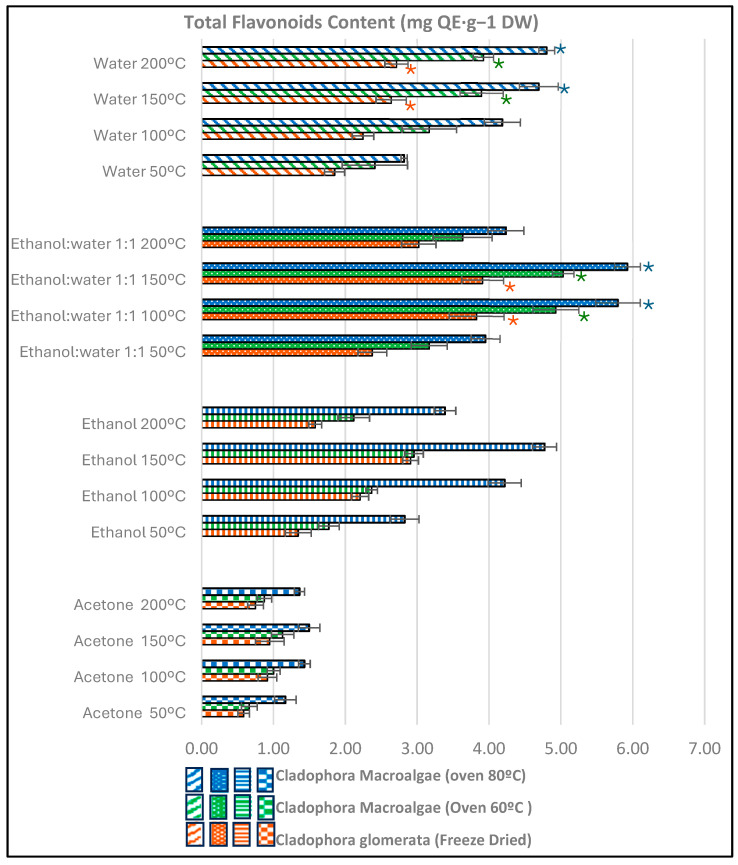
Mean concentration of flavonoids in *Cladophora glomerata* algae, freeze-dried, oven-dried (60 °C, 80 °C). No significant differences were observed between samples with the same color of asterisks (*) of every solvent, according to the Student–Newman–Keuls test, at *p* ≤ 0.05. QE—quercetin equivalent.

The total flavonoid content (TFC), expressed as mg quercetin/g dry algae, was quantified in PLE extracts using four solvents. Flavonoids were better isolated in the following order: E.W (1:1) > water > ethanol > acetone. The significantly highest flavonoid content was observed at 150 °C (*p* ≤ 0.05), although a very good level of flavonoids was also found at 100 °C (Figure 2).

The initial algae drying conditions are important since there is very little literature about the effects of drying on the green macroalgae bioactive flavonoids. Higher levels of TFC in oven-dried *Cladophora glomerata* samples compared to freeze-dried were observed for the four solvents and the four temperatures tested.

For example, TPC was 1.6 times higher in extracts of *Cladophora glomerata* oven-dried at 80 °C using PLE-150 °C with ethanol (3.39 QE/g d.w.) compared to freeze-dried samples under the same conditions (1.58 mg QE/g d.w.). Similar findings were reported by Uribe et al. [50], who found increases of 34% in oven-dried green algae in comparison with those freeze-dried.

The lowest flavonoid content was obtained after PLE with acetone (0.59–1.36 mg QE/g d.w.) and usually with all the organic solvents at 200 °C (Figure 2). The highest TFC value reached 5.92 mg QE/g d.w. in OD-80 °C *Cladophora glomerata* samples after PLE-150 °C with water–ethanol as medium–high-polarity solvent. There were no significant differences in comparison with PLE-100 °C (5.79 mg QE/g d.w).

Our proposed conditions using ethanol–water (1:1) as extraction solvent of PLE-150 °C in oven-dried algae at 80 °C showed highly advantageous flavonoid levels (5.92 mg QE/g) compared to 0.21–0.87 mg QE/g d.w.reported for ethanolic soxhlet extracts of several *Cladophora* species (*Glomerata*, *Rivularis*, and *Aegagropila*) [51]. In addition, the current results reveal higher extraction efficiency of flavonoids using PLE compared to 1.08–1.46 mg QE/g d.w. obtained by UAE, solid extraction, and MAE techniques [43,44] since PLE is advantageous for extracting heat-unstable flavonoids, whereas extraction methods that perform at relatively low temperatures over a long period of time result in lower flavonoid yields and higher levels of impurities.

It is noteworthy that the TFC thermal degradation after PLE-200 °C, except for *Cladophora glomerata* extracts obtained with water as solvent (4.80 mg QE/g d.w.), where flavonoids preservation by subcritical water isolation was observed, mainly in *Cladophora* samples oven-dried at 80 °C. Similar results described better isolation of algal flavonoids with aqueous solvents at high temperatures than with organic solvents such as hexane, chloroform, or methanol [52].

Therefore, our data suggest that the initial oven-dried treatment at 80 °C in conjunction with PLE at 150 °C using E:W (1:1) and the use of water at 200 °C for the isolation of flavonoids from *Cladophora glomerata* are both effective methods. In this sense, we have provided an effective condition for isolating flavonoids with subcritical water using safe and environmentally friendly alternatives.

### 3.3. Carotenoids and Chlorophyll a and b Content

The results showed that carotene and chlorophylls *a* and *b* were extracted more effectively when PLE was performed using ethanol > acetone > E.W (1:1) > water. In terms of temperature, the maximum extraction yields of these molecules were obtained for extracts reaching 150 °C and 100 °C during the PLE extraction cycles. Regarding the drying treatment prior to extraction, it can be observed that *Cladophora glomerata* algae samples OD-80 °C showed the highest mean values (Table 1). The chlorophyll content was found to be significantly different between the extracts.

The highest total carotenoid content (TCC) from *Cladophora glomerata* was quantified in ethanol and E.W (1:1) extracts (1.45 and 1.34 mg·g^−1^ d.w., respectively) using PLE-150 °C in samples previously dried in OD-80 °C. These results are in agreement with previous studies by Ernesta Tolpeznikaite et al. [40], where *Cladophora rupestris* carotenoids were found at 1.26 mg·g^−1^ d.w., and with other studies that mentioned a higher PLE efficiency for algae carotenoids extraction compared to maceration, soxhlet, and ultrasound-assisted [4,41,53]. The ethanol–water ratio is very important since Yarnpadee et al. reported lower values of carotenoid extraction in this alga when the ethanol percentage was less than 40% [49].

The PLE-50 °C provided the lowest extraction efficiency of TCC ranging from 0.09 to 0.16 mg·g^−1^ d.w., in agreement with 0.08 and 0.12 mg g^−1^ d.w. reported in samples of *Cladophora* spp. from several provenances [44,54].

It was found that the TCC content of the current *Cladophora glomerata* samples decreased significantly in freeze-dried and oven-dried extracts from PLE at 200 °C by degrading at high temperatures. This depletion was previously described based on the unstable character of carotenoids, which are sensitive to light, oxygen, and temperature [55].

Regarding the pigment, the better values of chlorophyll *a* and *b* were achieved in ethanolic extracts from PLE-150 °C using *Cladophora glomerata* samples previously oven-dried at 80 °C. (617.98, 520.10 µg·g^−1^ d.w., respectively). The higher isolation temperature seems to break a greater number of chloroplasts during the extraction process. In addition, PLE-150 °C can minimize the formation of pheophorbide, a toxic chlorophyll derivative since it has been described that heat treatment (>110 °C) deactivates the enzyme chlorophyllase [56,57] avoiding a decrease in the native forms from *Cladophora glomerata* samples. Our results are more effective than those found in the literature with other extraction methods: for instance, 138.44–200.79 and 119.05–308.43 μg·g^−1^ d.w. were reported for chlorophyll *a* and *b*, respectively, in *Cladophora glomerata* using acetone [30], 300 μg·g^−1^ d.w. by extraction with ethanol [44], as well as 430 and 120 μg·g^−1^ d.w., respectively, in samples collected from the Baltic sea [58].

In summary, one of the most interesting results of this study was the desirable effect of oven-dried at 80 °C, followed by PLE with ethanol at 150 °C, on the extraction of carotenoids and chlorophyll a and b from *Cladophora glomerata*. The above conditions are proposed as a highly efficient extraction of these natural compounds, which are currently regulated by the European Food Safety Authority (EFSA) [59]. This is due to the high commercial value of these compounds as food colors, feed supplements, additives, and functional ingredients. Additionally, the pharmaceutical industry utilizes these compounds for health purposes since chlorophyll and carotenoids exhibit significant antioxidant and anti-inflammatory effects.

### 3.4. Tocochromanols Determination

**Figure 3:**
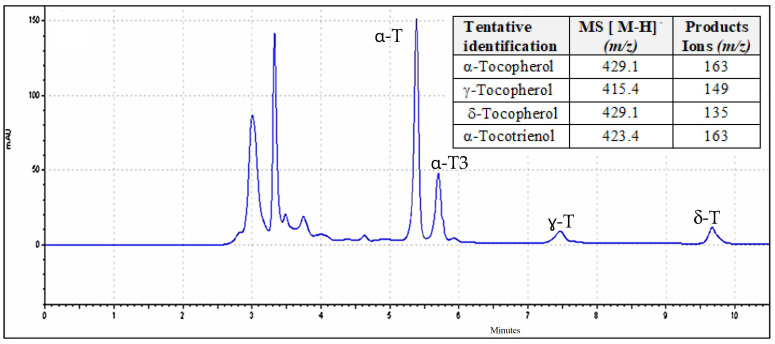
Chromatographic profile of tocopherols (T) and tocotrienols (T3) from *Cladophora glomerata*, previously oven-dried at 60 °C (using PLE at 150 °C). Mass spectrometry parameters for the isomer’s identification.

Vitamin E consists of four tocopherols (α-, β-, γ-, and δ-tocopherol) and four tocotrienols (α-, β-, γ-, and δ-tocotrienols), encompass a group of compounds referred to as tocochromanols or tools that possess potent antioxidant, anti-inflammatory, and neuroprotective properties since they are effective in preventing the propagation of peroxidation [60]. In the current work, three tocopherols (α-T, γ-T, and δ-T) and one tocotrienol (α-T3) were isolated for *Cladophora glomerata* (Figure 3). Their presence was confirmed by their [H-H]^−^ precursors and product ions. As far as the authors know, this is the first time that tocotrienols have been quantified in green macroalgae using the proposed methodology HPLC-UV.

The total content of tocochromanols in *Cladophora glomerata* extracts varied greatly depending on the previous drying treatment. Statistically significant differences (*p* ≤ 0.05) in OD samples (60 °C) in comparison with OD (80 °C) and FD were quantified (Table 2).

From a quantitative point of view, the highest amount of α-tocopherol (α-T) was found using acetone (361.41 µg·g^−1^) and ethanol (301.47 µg·g^−1^) both at PLE-150 °C in samples previously oven-dried (60 °C). Results obtained with PLE-100 °C also showed very good extraction yields for α-T (342.35, 252.36 µg·g^−1^ d.w., respectively) (Table 2). The current results widely exceed levels between 16.8 and 25.00 µg·g^−1^ d.w. reported in *Cladophora* spp. by solid–liquid extraction [4,60,61]. Overall, our results showed 8–14 times higher α-tocopherol than those obtained by classical isolation techniques.

In all cases, with the four solvents tested, it was observed that PLE-150 °C favors and increases the extractable tocols fraction. This is because increasing the temperature reduces the viscosity and increases the diffusivity, resulting in better solubility of the solute, as suggested by another research [62].

The worst response for tocols levels was obtained with PLE at 50 °C and 200 °C. The isolation temperature affected the extraction of vitamin E isomers and their thermal stability. α-T showed 6.81–8.01 µg·g^−1^ d.w. (in water extract), suggesting thermo-oxidation at high PLE temperatures, in agreement with other studies that estimated a loss of α-tocopherol of up to 87%, as the fatty acid peroxyl free radicals react with the phenolic hydrogen of the tocopherol molecule at 170–190 °C [63,64].

The average concentration of δ-tocopherol in *Cladophora glomerata* extracts using acetone at PLE-150 °C fluctuated from 18.52 to 29.58 μg·g^−1^. The results cannot be compared with other work in the literature because, to the knowledge of the authors, there are no published data on delta and gamma isomers in green macroalgae.

Concerning γ-tocopherol, similar levels were observed in acetone extracts of PLE-150 °C, from freeze-dried (23.73 μg·g^−1^) and oven-dried at 60 °C (28.14 μg·g^−1^) both statistically higher (*p* ≤ 0.05) than those quantified in oven-dried samples at 80 °C (16.85 μg·g^−1^) (Table 2). These values are higher than 15.00 μg·g^−1^ reported in *Monodopsis* sp. microalga [65]. Previous literature has described a higher capacity of γ-tocopherol than α-tocopherol to trap nitrogen oxide species and to inhibit cyclooxygenase activity, promoting antioxidant and anti-inflammatory effects [66,67]. For this reason, the remarkable γ-tocopherol levels found in dried *Cladophora glomerata* macroalgae can be considered a striking finding of this study, and they could enhance the antioxidant and anti-inflammatory properties of this macroalgae.

The α-tocotrienol (α-T3) level was found to be outstanding, ranging from 69.82 to 98.9 μg·g^−1^, in acetone extracts from *Cladophora glomerata* using PLE-150 °C. Remarkable amounts were also found in ethanol extracts between 34.25 and 83.14 μg·g^−1^. It has been reported that α-T3 provides neuroprotection in hippocampal neurons by exerting antioxidant properties [30]. In addition, α-T3 provides a decrease to the oxidative stress caused by ROS and RNS and possesses anti-inflammatory mechanisms such as suppression of inflammatory mediators such as IL-1, IL-6, IL-18, IL-8, and TNF-alpha in macrophages [31]. In this sense, our data are relevant because the presence of α-T3 represents a notable advantage in the composition of *Cladophora glomerata* algae, which may have therapeutic potential and could be utilized as a dietary supplement or in nutraceutical applications.

### 3.5. Antioxidant Activity of Cladophora Glomerata Extracts. Effects of Dried Treatments and PLE Conditions

The effect of drying on the antioxidant capacity of *Cladophora glomerata extracts*, not previously addressed, was assessed by DPPH, ABTS·+, and FRAP tests and expressed in terms of Trolox equivalent (TE) in Table 3.

The first conclusion that can be drawn is the positive effect of drying on the antioxidant power. The highest ABTS radical scavenging activities were found in oven-dried samples at 80 °C, showing significant and highest values (*p* < 0.05) than oven-dried at 60 °C and freeze-dried (Table 3). Results indicated that the choice of appropriate drying conditions prior to PLE extraction has a significant effect on the antioxidant activity. OD (80 °C) extracts with the highest levels of total polyphenols and total flavonoids, which play an important role in the adsorption and neutralization of free radicals, were also those that showed the highest antioxidant activity values.

The lowest ABTS, FRAP, and DPPH values were found in samples previously freeze-dried, revealing that lyophilization can preserve the initial compounds but does not approve the formation of new compounds or aglycones with antioxidant activity. Other green macroalgae also showed higher antioxidant activity in samples dried in a convection-drying oven compared to those dried using vacuum-drying [50]. Therefore, results suggest that an appropriate previous heat treatment of *Cladophora glomerata* samples could be used to enhance the antioxidant capacity of this macroalga.

Overall, in terms of PLE type of solvent and extraction temperature, it was found that all organic extracts obtained with acetone, ethanol, and even E:W (1:1) showed the highest antioxidant activities at PLE-150 °C. It is worth noting that among the solvents, the highest antioxidant activities were found with the green extract E:W (1:1) at PLE-150 °C.

In particular, the ABTS assay values observed in E:W extracts under these conditions were 59.15 mg TE/g dw (FD), 67.18 mg TE/g dw (OD 60 °C), and 81.96 mg TE/g dw (OD 80 °C). Similarly, DPPH values also reached the highest levels in E:W extracts at PLE-150 °C in OD samples at 80 °C, displaying 49.17 mg TE/g dw. An identical behavior was found for the FRAP antioxidant values with 2.64 TE/g dw in E:W extracts previously oven-dried at 80 °C. Our increased antioxidant activity values in heated samples should be due to the formation of new low-molecular-weight phenols (as a result of the endogenous algae enzymes), as well as the probable transformation of glycoside forms into their corresponding free aglycones, which are highly soluble in ethanol–water mixtures, both chemical behaviors previously proved by other authors addressing similar studies [68,69,70,71].

**Figure 4:**
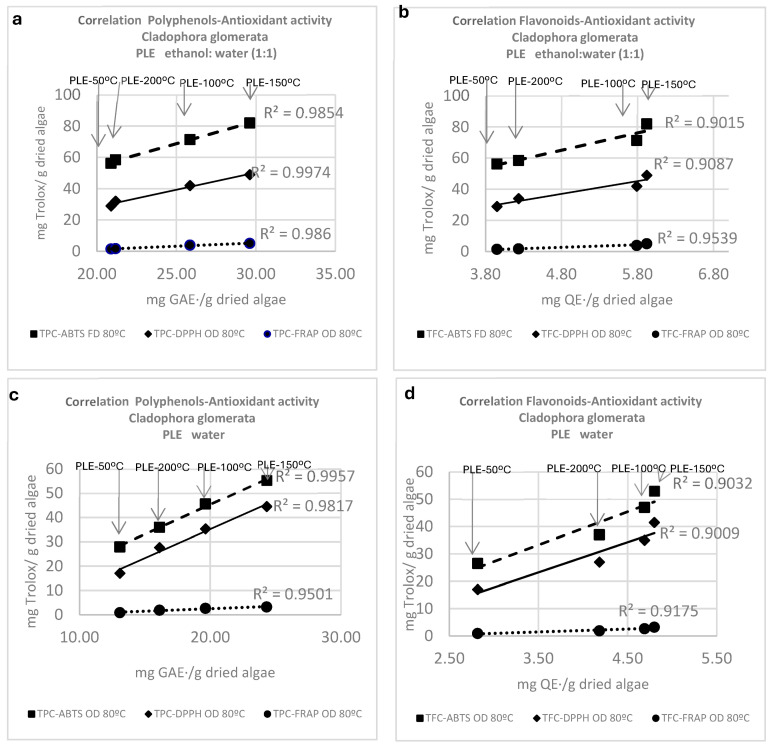
Correlation between total phenols content (TPC) and total flavonoids content (TFC) with the antioxidant activity (DPPH, FRAP, and ABTS assay) in *Cladophora glomerata* algae oven-dried at 80 °C and submitted to PLE extraction at 50 °C, 100 °C, 150 °C, and 200 °C using ethanol–water (1:1) (**a**,**b**) and water (**c**,**d**).

The correlation coefficients between total polyphenol content (TPC) and total flavonoid content (TFC) with the ABTS, FRAP, and DPPH assays are shown in Figure 4. A striking and linear relationship was observed for E:W extracts (1:1) and water extracts for the three drying conditions and for the PLE solvents and temperatures analyzed, with R^2^ correlation coefficients of up to 0.9. For example, for the TPC-ABTS, the R^2^ correlation coefficient was 0.9854 and 0.9957 for both environmentally friendly solvents, while R^2^ was 0.915 and 0.9032 for the TFC-ABTS. Carotenoids, chlorophyll, and tocopherol isomers did not show a linear correlation with respect to antioxidant activity, meaning that TPC and TFC were the main factors accounting for the antioxidant activity of the *Cladophora glomerata* extracts.

One of the most interesting findings of the current experiment was the significantly higher values (*p* ≤ 0.05) observed in the ABTS, DPPH, and FRAP assays in aqueous extracts of *Cladophora glomerata* obtained at PLE-200 °C (subcritical conditions) and PLE-150 °C.

Aqueous extracts at PLE-200 °C displayed ABTS values of 61.34, 50.60, and 45.33 mg TE/g in OD (80 °C), OD (60 °C), and FD samples, respectively. DPPH assay values ranged from 20.17 to 35.29 mg TE/g and FRAP reduction activity from 1.65 to 3.20 mg TE/g using water as the extractant. The results observed in aqueous extracts of oven-dried samples at 80 °C may be due to hydrolytic degradation of the glycoside forms of phenolic compounds, especially when water is used (in whole or in part) as the extractant solvent, as previously observed by other authors [72,73].

Subcritical water extraction enhanced cell wall breakdown, facilitating the release and extraction of antioxidants, an effect that increased with temperature. Other authors have worked with this technique; for instance, Suthasinee Yarnpakdee et al. [49] reported ABTS values of 130 µM/g in 100% aqueous extract of *Cladophora glomerata*, which is equivalent, taking into account the molecular weight of Trolox, to 32.5 mgTE/g. Korzeniowska et al. [43] listed ABTS, DPPH, and FRAP levels in aqueous extracts from MAE and UAE; however, data are between 1.9 and 6 times lower than those found here.

Therefore, oven-drying at 80 °C together with PLE with ethanol–water (1:1) at 150 °C or with water alone at 200 °C seem to be the most effective extraction conditions to increase the antioxidant activity in extracts from *Cladophora glomerata*, and they can be a suitable technique to obtain natural extracts with outstanding antioxidant potential.

Considering that methanol cannot be used for preparations used in the food industry, our ethanol–water and aqueous eco-friendly extracts can be a more suitable alternative.

These are excellent results since the current technological conditions have allowed us to obtain aqueous extracts from the macroalgae *Cladophora glomerata*, which have an exceptional antioxidant potential that could be translated into the cosmetic, pharmaceutical, and agricultural industries. What is more, they can constitute important and health-promoting components in products that protect against oxidative stress.

### 3.6. Formulation of Nanoemulsion Systems from Cladophora glomerata

The selection of the different oily cores, i.e., Miglyol © or soybean oil, as well as the extraction method, displayed a minor effect on the physicochemical properties of the *Cladophora glomerata* nanoemulsions (NEs). Three PLE extracts were chosen for this study based on their highest levels of bioactive compounds and antioxidant potential. The chosen extracts were W:E-150 °C, W:E-100 °C, and water-200 °C from macroalgae oven-dried at 80 °C obtained with PLE.

**Figure 5:**
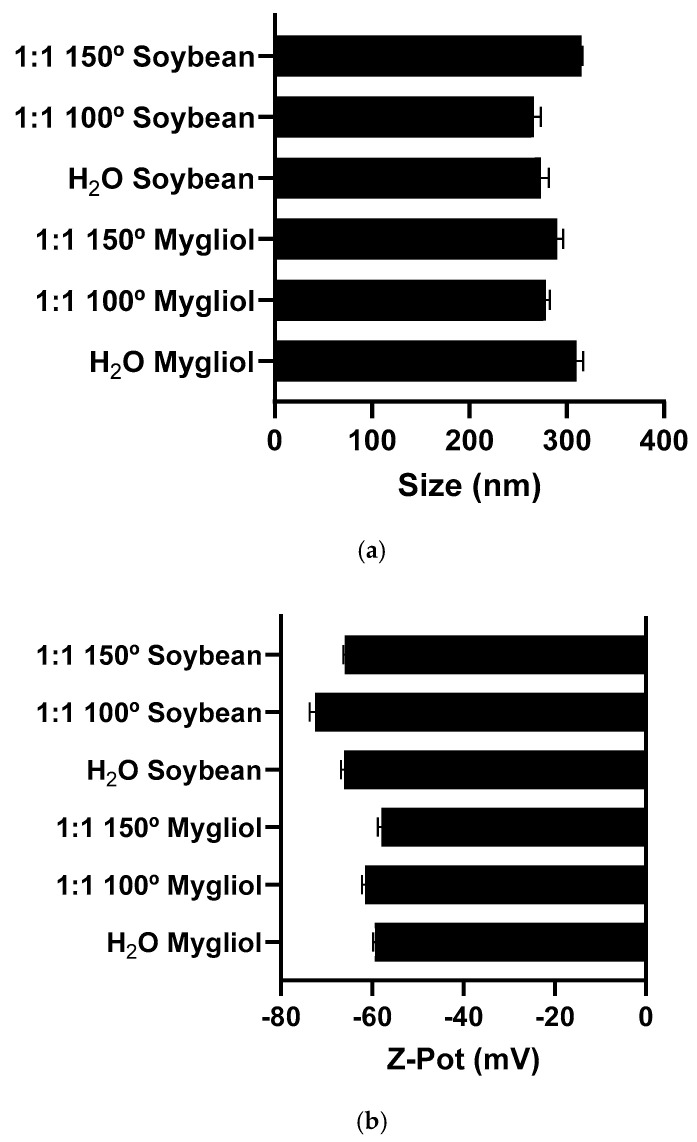
Hydrodynamic mean size of the nanoemulsions formulated either with Mygliol © or Soybean oils with the three different *Cladophora glomerata* extracts (**a**). Z-Potential of the nanoemulsions formulated either with Mygliol © or Soybean oils with 3 different *Cladophora glomerata* extracts (**b**). 1:1 150°: PLE *Cladophora* G. extracts obtained with ethanol–water (1:1) at 150 °C 1:1 100°: PLE *Cladophora* G. extracts obtained with ethanol–water (1:1) at 100 °C 1:1 100°: PLE *Cladophora* G. extracts obtained with water at 200 °C.

As shown in Figure 5a, with both oils, it was feasible to formulate NEs with a hydrodynamic mean size in the 300 nm range (from 275 up to 310 nm) and low PDI in both cases (<0.2). Both oils led to formulations with similar negative ζ-Potential. The use of the Miglyol © led to a superficial charge of around −60 mV, while the use of soybean oil resulted in the formation of an oily droplet with a superficial charge of around −70 mV (see Figure 5b). In both cases, the negative superficial charge of the NEs can be attributed to the composition of the shell of the oily droplets, which is mainly formed by lecithin, a mixture of phospholipids that contains negatively charged components.

Considering that the bioactive compounds found in *Cladophora glomerata* can be oxidized by external factors, their formulation as extract-enriched NEs will protect these components from premature oxidation during the formulation and storage of the extract [25,32]. In this sense, the formulation of the *Cladophora glomerata* extracts as NEs will help to preserve the health benefits of this green macroalgae.

### 3.7. In Vitro Studies in Macrophage Cell Line J774. Intracellular ROS Levels Measurements

Once the capacity of the different extracts for the formulation of nanosystems was demonstrated, the following studies focused on nanoemulsions formulated either with Miglyol © or soybean with the *Cladophora* extracts. Among the three extracts in the above section, ethanol–water (1:1) at 150 °C was selected for the in vitro study in the J774 macrophage cell line. Although the different extracts led to nanoemulsions with similar physicochemical properties, this choice was motivated by the fact that this extract presented the highest diversity and concentration of bioactive compounds and showed the highest antioxidant potential of all the extracts obtained in this work.

**Figure 6:**
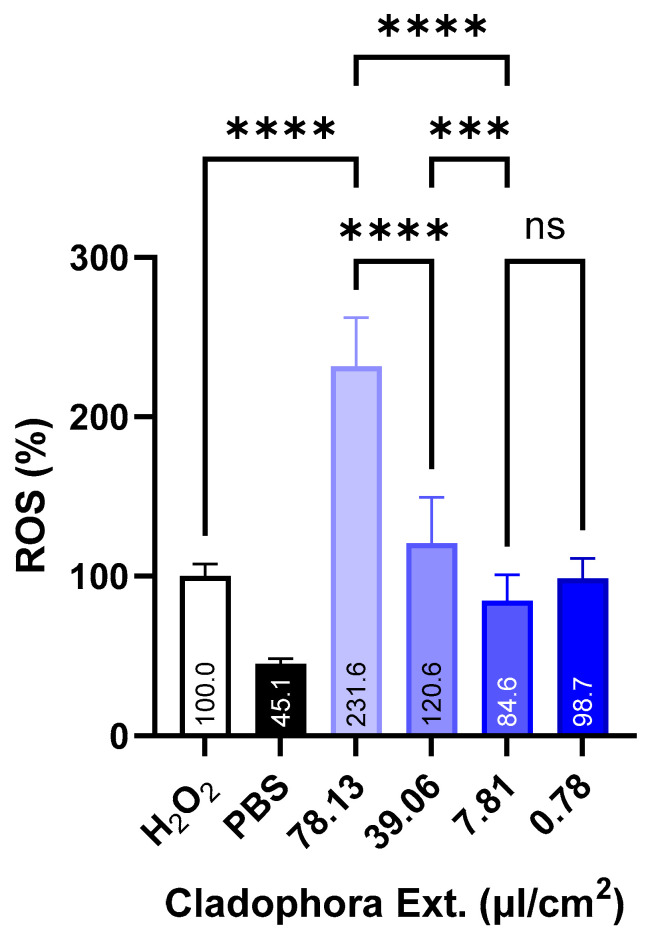
Antioxidant effect of the three free extracts from *Cladophora glomerata* at different concentrations on the intracellular ROS generation in J774.1 macrophage cells after oxidative stress induced by H_2_O_2_. The ROS percentages were calculated considering 123.5 mM of H_2_O_2_ in relation to 100% ROS. Significant difference *p* ≤ 0.001 (***), *p* ≤ 0.0001 (****).

Bearing this in mind, we selected the macrophage J774.1 to test the antioxidant effect of the free and encapsulated *Cladophora glomerata* extract. The first set of experiments was focused on the determination of the optimal concentration of extract to minimize the intracellular ROS levels in the macrophages after incubation with H_2_O_2_. As shown in Figure 6, high concentrations of *Cladophora glomerata* extracts led to a remarkable prooxidative effect, which may be due to the strong antioxidant effect displayed by this extract [74]. This fact is previously well documented as an “antioxidant paradox” [75], referring to contradictory observations where benefits are not always observed in clinical studies and in vivo systems. Antioxidants in high concentrations may cause reductive stress and manifest contradicting impacts of antioxidant treatment based on the redox balance to support physiological levels of ROS production for adequate cell function [76].

By reducing the extract concentration, it was possible to ameliorate the prooxidative effect, reaching a ROS % around 85% at a concentration of 7.81 μL/cm^2^. Interestingly, the reduction in the extract concentration even more (down to 0.78 μL/cm^2^) resulted in an increment of the ROS levels in the macrophage cells (∼99%). A sign that we were moving away from the optimum extract concentration. Based on these results, we selected the 7.81 μL/cm^2^ to analyze the effect of the NEs on the potential of this extract.

Antioxidant effect of the free extract from *Cladophora glomerata* at different concentrations on the intracellular ROS generation in J774.1 macrophage cells after oxidative stress induced by H_2_O_2_

**Figure 7:**
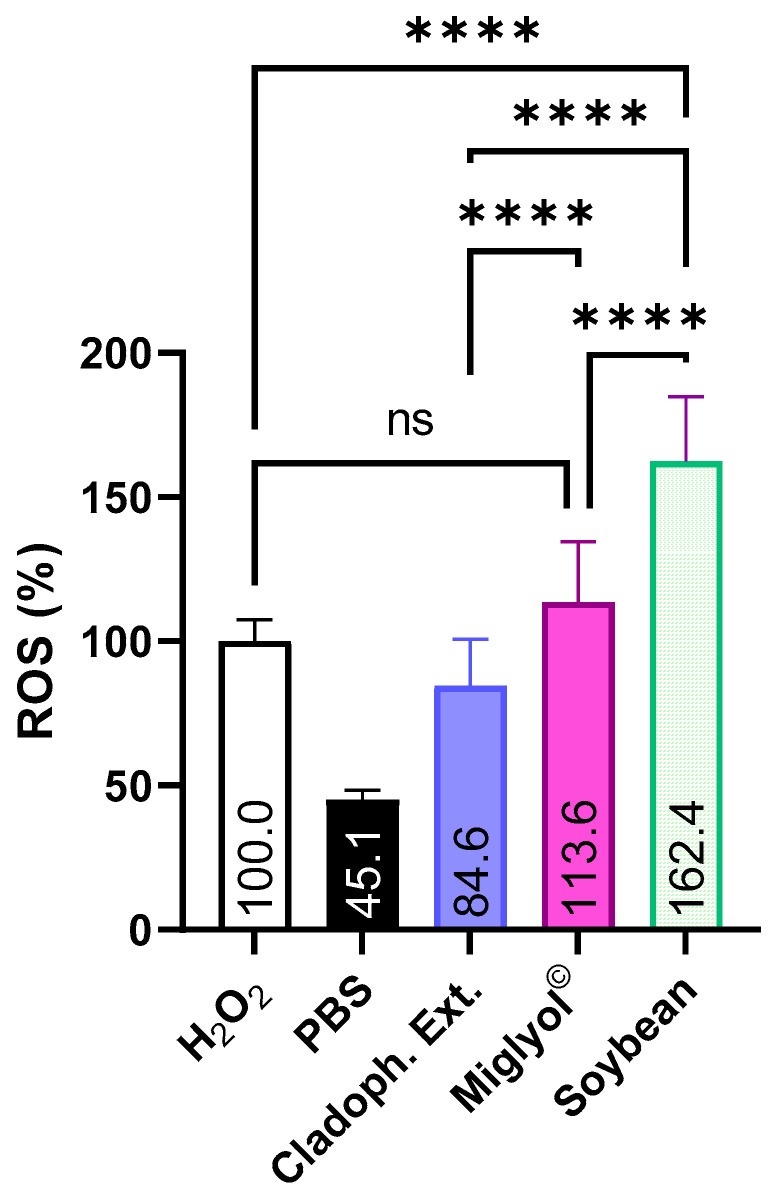
Effect of the type of nanoemulsion from *Cladophora glomerata* on the intracellular ROS in J774.1 macrophage cells after oxidative stress induced by H_2_O_2_. Cladoph. Ext: free *Cladophora glomerata* extracts from hydroalcoholic PLE-150 °C Miglyol ©: nanoemulsion of *Cladophora glomerata* using Miglyol Soybean: nanoemulsion of *Cladophora glomerata* using soybean oil. *p* ≤ 0.0001 (****).

As can be seen in Figure 7, the incorporation of the extract in NEs resulted in an intracellular ROS level significantly higher than those achieved by the free extract. This response of the macrophages can be attributed to a higher intracellular concentration of the *Cladophora glomerata* extract once formulated as NEs in comparison with the free extract dispersed in the cell medium [25,77].

Interestingly, the intracellular ROS level was clearly dependent on the nature of the oily core of the NEs [35]. While the Miglyol © core did not show significant differences with respect to the H_2_O_2_ control, NEs formulated with soybean oil displayed a significant prooxidative effect in comparison with both the H_2_O_2_ control and the NEs formulated with Miglyol ©. This can be attributed to the synergic effect of both the extract matrix and the antioxidant nature of the soybean oily core.

To obtain a better insight into the role of the formulated NEs from *Cladophora glomerata* extracts on the cellular internalization of the antioxidants, the last set of experiments was focused on the modulation of the macrophage–NE interaction. Bearing this in mind, we incorporated Pluronic © PF 68 at a concentration of 5% (*w*/*v*) onto the surface of both Miglyol © and soybean NEs. It is well known that this surfactant modulates the interaction of nanostructures and cells, reducing their internalization [33]. In this context, both NEs showed a reduction in the ROS percentage in the range of 22–24% (from 113.6 down to 87.8 and from 162.4 down to 124.4 for the Miglyol © and soybean NEs, respectively).

Altogether, these results clearly show that *Cladophora glomerata* extracts present great potential as antioxidant agents. However, the high antioxidant power of the extract requires the rational design of the *Cladophora glomerata* formulation. In this line, our results clearly show that the appropriate selection of excipients and their right formulation in the form of NEs may pave the way for the use of this type of therapeutic agent and ensure the proper interaction of the antioxidants with the target cells in order to maximize their therapeutic effect while avoiding the potential undesirable pro-oxidant effect of this potent extract.

## 4. Conclusions

We can affirm that an appropriate drying treatment, together with the optimization of PLE conditions, has a desirable strong influence, providing *Cladophora glomerata* extracts with outstanding levels of antioxidant bioactive compounds.

One of the main conclusions of this research suggests that the choice of oven-drying at 80 °C may result in a more appropriate drying method to retain polyphenols and flavonoids from *Cladophora glomerata* macroalga than oven-drying at 60 °C and freeze-drying. The PLE technique using ethanol–water (1:1) at 150 °C allowed obtaining *Cladophora glomerata* extracts with the highest TPC and TFC, avoiding their thermal degradation. PLE extraction using water at subcritical conditions (200 °C) displayed similar results. In this sense, the drying conditions and the optimized parameters of the advantageous PLE technology proposed here are highly effective in performing *Cladophora glomerata* extracts enriched in TPC and TFC using environmentally friendly solvents as an excellent alternative, with better results than those available in another research using soxhlet, UAE, and MAE.

*Cladophora glomerata* proved to be an important source of carotenoids and chlorophyll pigments. The oven-drying at 80 °C followed by PLE using ethanol as extraction solvent at 100 °C resulted in the most efficient conditions for the simultaneous extraction of the carotenoids and pigments, avoiding the degradation that leads to a decrease in the macroalga native forms.

From this study, it can be concluded that initial drying affects tocopherol and tocotrienol levels differently than the other compounds. Oven-drying at 60 °C and freeze-drying have a positive effect on preserving the tocochromanols. PLE with acetone at 150 °C is a suitable technique that allows an increase in the α-tocopherol levels from *Cladophora glomerata* compared to other procedures described in the literature. In addition, to the authors’ knowledge, γ-tocopherol, δ-tocopherol, and α-tocotrienol, associated with a reduction in oxidative stress, are described for the first time in these green macroalgae.

The highest antioxidant values of ABTS, DPPH, and FRAP were observed in oven-dried samples at 80 °C followed by PLE with ethanol–water (1:1) at 150 °C as well as in aqueous extracts at PLE-200 °C (subcritical conditions), concluding that these conditions can be considered a tool to increase the global antioxidant potential of *Cladophora glomerata* extracts. The strong correlation between TPC and TFC, with the greatest antioxidant assay values, demonstrates the important role of these compounds in the adsorption and neutralization of free radicals.

The screening of samples identified hydroalcoholic PLE green extracts from *Cladophora glomerata* algae as the best choice in terms of enriched-bioactive constituents and antioxidant potential. They were selected for the nanotechnology approaches, allowing the formulation of antioxidant-loaded NEs stabilized with lecithin.

The successful design of NEs formulated with hydroalcoholic extracts from *Cladophora glomerata* at a concentration of 7.81 μL/cm^2^ provided protection against oxidative stress, alleviating the ROS percentage around 85% in a macrophages cell line J774.1, incubated with H_2_O_2_, without generating toxic effect for the cells.

This study also evidences excellent results in enhancing the antioxidant activity of *Cladophora glomerata* macroalgae extracts since NEs formulated with Miglyol © and soybean allowed the modulation of the macrophage–NE interaction, achieving a reduction in ROS levels in the range of 22–24%.

Therefore, innovative perspectives associated with green macroalgae valorization can be developed. New approaches using nanotechnology platforms together with low-cost PLE extracts from *Cladophora glomerata* using environmentally friendly solvents represent a promising strategy for future applications against oxidative stress in various fields, including biomedicine, cosmetics, functional foods, and nutraceuticals.

## Figures and Tables

**Table 1:** Mean concentration and relative standard deviations of total carotenoids content (TCC) (mg/g dry weight) and chlorophyll (Chlp) *a* and *b* (μg/g dry weight) in *Cladophora glomerata* macroalgae, previously freeze-dried, oven-dried (60 °C), and oven-dried (80 °C).

	*Cladophora glomerata* (Freeze-Dried)						*Cladophora glomerata* (Oven-Dried 60 °C)						*Cladophora glomerata* (Oven-Dried 80 °C)					
	chlp *a* µg·g^−1^	RSD	chl *b* µg·g^−1^	RSD	TCC mg·g^−1^	SD	chlp *a* µg·g^−1^	RSD	chl *b* µg·g^−1^	RSD	TCC mg·g^−1^	RSD	chlp *a* µg·g^−1^	RSD	chl *b* µg·g^−1^	RSD	TCC mg·g^−1^	RSD
**Acetone 50 °C**	48.18	(3.63)	37.07	(7.29)	0.09	(0.02)	53.49	(6.74)	43.74	(5.52)	0.11	(0.02)	89.40	(8.70)	72.33	(6.54)	0.10	(0.01)
**Acetone 100 °C**	68.72	(2.90)	52.86	(6.43)	0.66	(0.22)	76.28	(2.73)	62.38	(2.23)	0.85	(0.08)	156.75	(7.67)	131.92	(7.61)	0.42	(0.12)
**Acetone 150 °C**	73.62	(3.80)	56.63	(7.50)	0.71	(0.13)	81.72	(6.76)	66.82	(5.54)	1.10	(0.15)	167.93	(8.95)	141.33	(8.88)	0.60	(0.14)
**Acetone 200 °C**	55.57	(3.25)	42.75	(6.84)	0.33	(0.12)	61.69	(6.59)	50.44	(5.41)	0.40	(0.12)	126.76	(8.17)	106.69	(5.14)	0.40	(0.13)
**Ethanol 50 °C**	110.83 ^a^	(3.90)	85.25 ^b^	(7.62)	0.13 ^a^	(0.01)	252.80 ^a^	(7.25)	212.76 ^a^	(5.94)	0.14 ^a^	(0.02)	305.27 ^a^	(9.09)	245.87 ^a^	(9.02)	0.16 ^a^	(0.02)
**Ethanol 100 °C**	158.05 ^b^	(4.13)	121.58 ^b^	(7.89)	0.73 ^b^	(0.33)	360.53 ^b^	(2.73)	303.43 ^b^	(2.23)	0.80 ^b^	(0.13)	576.85 ^b^	(9.41)	485.48 ^b^	(8.11)	1.32 ^b^	(0.15)
**Ethanol 150 °C**	169.33 ^b^	(4.77)	130.25 ^b^	(4.76)	0.80 ^b^	(0.21)	386.24 ^c^	(6.38)	325.06 ^c^	(5.23)	1.25 ^c^	(0.14)	617.98 ^c^	(5.68)	520.10 ^c^	(6.57)	1.45 ^b^	(0.24)
**Ethanol 200 °C**	127.82 ^c^	(3.68)	98.32 ^b^	(7.35)	0.61 ^c^	(0.22)	291.55 ^d^	(6.92)	245.38 ^d^	(5.68)	0.76 ^b^	(0.08)	466.49^d^	(8.78)	392.60 ^d^	(5.64)	1.05 ^c^	(0.23)
**E:W (1:1) 50 °C**	97.92 ^a^	(3.70)	75.32 ^a^	(7.38)	0.06 ^a^	(0.01)	211.11 ^a^	(3.27)	185.66 ^a^	(2.68)	0.07 ^a^	(0.01)	234.38 ^a^	(8.80)	198.21 ^a^	(8.74)	0.15 ^a^	(0.06)
**E:W (1:1) 100 °C**	148.66 ^b^	(3.55)	114.35 ^b^	(7.20)	0.25 ^b^	(0.14)	339.09 ^b^	(6.68)	285.38 ^b^	(6.48)	0.30 ^b^	(0.17)	542.54 ^b^	(8.59)	456.61 ^b^	(8.53)	1.17 ^b^	(0.11)
**E:W (1:1) 150 °C**	159.89 ^b^	(3.76)	122.99 ^b^	(7.45)	0.36 ^c^	(0.23)	364.71 ^c^	(5.89)	306.95 ^b^	(4.83)	0.44 ^c^	(0.13)	583.54 ^b^	(8.89)	491.11 ^c^	(8.83)	1.34 ^b^	(0.14)
**E:W (1:1) 200 °C**	113.95 ^c^	(4.15)	87.65 ^c^	(7.91)	0.24 ^b^	(0.15)	259.91 ^d^	(6.87)	218.75 ^c^	(5.63)	0.29 ^b^	(0.12)	315.17 ^c^	(9.44)	249.25 ^d^	(9.25)	0.55 ^c^	(0.08)
**Water 50 °C**	22.17	(3.35)	17.05	(6.96)	tr		24.60	(5.45)	20.12	(3.24)	tr		67.41	(8.31)	56.74	(4.25)	tr	
**Water 100 °C**	31.61	(3.85)	24.32	(7.56)	tr		35.09	(6.98)	28.69	(2.98)	tr		94.14	(9.02)	80.91	(8.95)	tr	
**Water 150 °C**	33.87	(3.63)	26.05	(4.76)	tr		37.59	(7.23)	30.74	(2.65)	tr		105.00	(5.68)	86.68	(5.64)	tr	
**Water 200 °C**	31.56	(3.90)	23.66	(3.57)	tr		35.38	(4.36)	28.20	(3.58)	tr		98.75	(4.26)	84.43	(4.23)	tr	

a–d: Significant differences (*p* ≤ 0.05) according to the Student–Newman–Keuls test at *p* ≤ 0.05 for samples with the best isolation results. E:W: ethanol–water (1:1 *v*/*v*); tr: traces; n.d: not detected.

**Table 2:** Vit E. Mean values and relative standard deviations of tocopherols (T) and tocotrienols (T3) from *Cladophora glomerata*.

µg·g^−1^ D.W.	*Cladophora glomerata* (Freeze-Dried)								*Cladophora glomerata* (Oven-Dried 60 °C)								*Cladophora glomerata* (Oven-Dried 80 °C)							
	αT		αT3		γT		δT		αT		αT3		γT		δT		αT		αT3		γT		δT	
**Acetone 50 °C**	212.65 ^a^ *	(6.05)	67.01	(3.05)	16.73	(0.15)	18.44	(1.04)	218.04 ^a^ *	(7.56)	68.78	(3.06)	17.19	(0.25)	19.26	(1.03)	150.75 ^b^ *	(11.14)	45.59	(2.05)	9.70	(1.62)	10.13	(0.07)
**Acetone 100 °C**	337.65 ^a^ * ^Ϯ^	(8.69)	88.94	(1.58)	22.24	(0.52)	22.56	(1.42)	342.35 ^a^ * ^Ϯ^	(6.28)	90.24	(2.96)	22.49	(0.63)	23.91	(0.63)	253.60 ^b^ *	(8.59)	61.29	(2.36)	11.68	(2.08)	12.29	(0.11)
**Acetone 150 °C**	358.05 ^a^ * ^Ϯ Ϯ^	(11.97)	96.99	(2.51)	23.73	(0.14)	26.50	(0.69)	361.41 ^a^ * ^Ϯ Ϯ^	(16.54)	98.90	(0.78)	28.14	(1.98)	29.58	(1.98)	284.00 ^b^ *	(13.67)	69.82	(1.36)	16.85	(0.05)	18.52	(0.85)
**Acetone 200 °C**	203.54 ^a^ *	(5.44)	65.87	(1.74)	16.81	(0.41)	18.84	(0.99)	207.93 ^a^ *	(5.20)	67.26	(1.65)	17.05	(1.01)	19.02	(0.08)	143.01 ^b^ *	(9.61)	44.18	(1.09)	9.40	(0.17)	9.82	(0.04)
**Ethanol 50 °C**	147.24 ^a^ **	(8.85)	56.39	(2.22)	12.05	(0.04)	15.21	(0.08)	152.47 ^a^ **	(9.80)	57.31	(1.60)	12.11	(1.19)	15.77	(1.12)	99.60 ^b^ **	(9.36)	20.79	(0.35)	8.39	(0.32)	12.35	(0.21)
**Ethanol 100 °C**	252.12 ^a^ ** ^Ϯ^	(3.23)	71.04	(1.54)	15.82	(0.16)	20.04	(1.01)	252.36 ^a^ ** ^Ϯ Ϯ^	(11.90)	73.55	(2.16)	14.11	(1.26)	20.92	(0.71)	184.51 ^b^ **	(8.36)	27.64	(0.27)	10.69	(0.08)	15.11	(0.32)
**Ethanol 150 °C**	295.22 ^a^ ** ^Ϯ Ϯ^	(9.13)	80.20	(1.11)	19.09	(0.23)	26.32	(0.99)	301.47 ^a^ ** ^Ϯ Ϯ^	(8.87)	83.14	(2.27)	19.31	(0.07)	25.69	0.58)	219.24 ^b^ **	(7.05)	34.25	(0.20)	14.62	(0.17)	13.74	(0.29)
**Ethanol 200 °C**	112.65 ^a^ **	(9.92)	50.67	(2.87)	11.71	(0.18)	16.21	(0.07)	115.93 ^a^ **	(4.26)	53.07	(1.36)	12.07	(1.07)	17.77	(0.18)	74.90 ^b^ **	(10.54)	15.09	(0.16)	9.91	(0.05)	10.49	(0.09)
**E:W (1:1) 50 °C**	60.21 ^a^	(8.82)	9.25	(0.13)	tr		tr		63.25 ^a^	(5.13)	10.54	(0.07)	tr		tr		33.76 ^b^	(6.54)	tr		tr		tr	
**E:W (1:1) 100 °C**	84.58 ^a^	(3.33)	14.20	(0.21)	tr		tr		85.21 ^a^	(2.92)	14.99	(0.11)	tr		tr		52.43 ^b^	(8.82)	tr		tr		tr	
**E:W (1:1) 150 °C**	89.36 ^a^	(4.05)	14.89	(0.07)	tr		tr		92.19 ^a^	(2.11)	15.36	(0.29)	tr		tr		55.96 ^b^	(6.54)	tr		tr		tr	
**E:W (1:1) 200 °C**	53.71 ^a^	(2.13)	8.19	(0.12)	tr		tr		55.63 ^a^	(3.03)	8.95	(0.31)	tr		tr		55.65 ^b^	(7.72)	tr		tr		tr	
**Water 50 °C**	6.00 ^a^	(0.95)	n.d.		n.d.		n.d.		6.36 ^a^	(0.03)	n.d.		n.d.		n.d.		4.10 ^b^	(0.33)	n.d.		n.d.		n.d.	
**Water 100 °C**	12.52 ^a^	(1.02)	n.d.		n.d.		n.d.		13.10 ^a^	(0.51)	n.d.		n.d.		n.d.		9.64 ^b^	(0.41)	n.d.		n.d.		n.d.	
**Water 150 °C**	16.87 ^a^	(1.32)	n.d.		n.d.		n.d.		17.28 ^a^	(0.97)	n.d.		n.d.		n.d.		12.36 ^b^	(0.87)	n.d.		n.d.		n.d.	
**Water 200 °C**	8.01 ^a^	(0.25)	n.d.		n.d.		n.d.		8.89 ^a^	(2.21)	n.d.		n.d.		n.d.		6.81 ^b^	(1.25)	n.d.		n.d.		n.d.	

E:W: ethanol–water (1:1 *v*/*v*); Tr: traces; n.d: not detected. a, b: significant differences (*p* ≤ 0.05) according to the Student–Newman–Keuls test (SNK) depending on the type of sample drying (FD, oven-dried at 60 °C, 80 °C). *, **: significant differences (*p* ≤ 0.05) according to the SNK test at *p* ≤ 0.05, depending on the type of solvent in the three types of drying. Ϯ, Ϯ Ϯ: significant differences (*p* ≤ 0.05) according to the SNK test at *p* ≤ 0.05, depending on the solvent temperature: comparing 100 °C and 150 °C.

**Table 3:** Antioxidant activity (mg Trolox·g^−1^ dry weight) and relative standard deviations for freeze-dried at oven-dried *Cladophora glomerata* samples.

mg Trolox·g^−1^	*Cladophora glomerata* (Freeze-Dried)						*Cladophora glomerata* (Oven-Dried 60 °C)						*Cladophora glomerata* (Oven-Dried 80 °C)					
	ABTS		DPPH		FRAP		ABTS		DPPH		FRAP		ABTS		DPPH		FRAP	
**Acetone 50 °C**	7.34 ^a^	(3.63)	5.12 ^a^	(2.54)	0.05 ^a^	(0.01)	8.47 ^a^	(6.74)	6.09 ^a^	(5.52)	0.05 ^a^	(0.02)	9.96 ^a^	(8.70)	8.78 ^a^	(6.54)	0.07 ^a^	(0.01)
**Acetone 100 °C**	11.29 ^b^	(2.90)	8.28 ^b^	(2.54)	0.17 ^b^	(0.02)	13.69 ^b^	(2.73)	8.01 ^ab^	(2.23)	0.21 ^b^	(0.02)	15.85 ^b^	(7.67)	14.36 ^b^	(7.61)	0.29 ^b^	(0.02)
**Acetone 150 °C**	12.32 ^b^	(3.80)	9.14 ^b^	(2.65)	0.21 ^b^	(0.03)	17.06 ^c^	(6.76)	9.02 ^b^	(5.54)	0.30 ^c^	(0.06)	18.57 ^b^	(8.95)	19.36 ^c^	(8.88)	0.41 ^c^	(0.04)
**Acetone 200 °C**	9.09	(3.25)	6.34 ^ab^	(4.94)	0.11 ^c^	(0.02)	11.41 ^b^	(6.59)	7.17 ^ab^	(5.41)	0.18 ^b^	(0.02)	13.66 ^a^	(8.17)	13.02 ^b^	(5.14)	0.21 ^b^	(0.03)
**Ethanol 50 °C**	12.35 ^a^	(3.90)	8.27 ^a^	(2.44)	0.09 ^a^	(0.01)	13.86 ^a^	(7.25)	11.36 ^a^	(5.94)	0.11 ^a^	(0.02)	25.56 ^a^	(9.09)	14.22 ^a^	(9.02)	0.18 ^a^	(0.02)
**Ethanol 100 °C**	17.45 ^b^	(4.13)	12.57 ^b^	(0.84)	0.24 ^b^	(0.09)	22.24 ^b^	(2.73)	13.69 ^b^	(2.23)	0.31 ^b^	(0.03)	35.93 ^b^	(9.41)	28.95 ^b^	(8.11)	0.74 ^b^	(0.11)
**Ethanol 150 °C**	22.33 ^c^	(4.77)	16.35 ^c^	(2.16)	0.35 ^c^	(0.07)	33.38 ^c^	(6.38)	17.23 ^c^	(5.23)	0.57 ^b^	(0.03)	45.56 ^c^	(5.68)	32.16 ^b^	(6.57)	0.85 ^b^	(0.04)
**Ethanol 200 °C**	15.68 ^b^	(3.68)	11.03 ^b^	(3.23)	0.19 ^d^	(0.02)	17.13 ^a^	(6.92)	12.01 ^ab^	(5.68)	0.22 ^ab^	(0.02)	33.41 ^b^	(8.78)	22.14 ^c^	(5.64)	0.52 ^c^	(0.05)
**E:W (1:1) 50 °C**	37.20 ^a^	(3.70)	17.11 ^a^	(6.16)	0.8 ^a^	(0.02)	41.32 ^a^	(3.27)	23.17 ^a^	(2.68)	1.02 ^a^	(0.08)	46.21 ^a^	(8.80)	29.08 ^b^	(8.74)	1.44 ^a^	(0.14)
**E:W (1:1) 100 °C**	50.86 ^b^	(3.55)	25.00 ^b^	(0.43)	1.85 ^b^	(0.13)	55.67 ^b^	(6.68)	34.00 ^b^	(5.48)	2.36 ^b^	(0.13)	71.33 ^b^	(8.59)	42.10 ^b^	(8.53)	3.90 ^b^	(0.15)
**E:W (1:1) 150 °C**	59.15 ^c^	(3.76)	30.04 ^c^	(1.53)	2.65 ^c^	(0.25)	67.18 ^v^	(5.89)	42.06 ^c^	(4.83)	3.58 ^c^	(0.19)	81.96 ^c^	(8.89)	49.17 ^b^	(8.83)	5.00 ^b^	(0.21)
**E:W (1:1) 200 °C**	48.10 ^b^	(4.15)	22.50 ^b^	(1.65)	1.55 ^b^	(0.20)	52.82 ^b^	(6.87)	30.60 ^b^	(5.63)	2.02 ^b^	(0.11)	62.50 ^d^	(9.44)	32.02 ^a^	(6.25)	1.70 ^a^	(0.16)
**Water 50 °C**	22.92 ^a^	(3.35)	14.12 ^a^	(4.43)	0.5 ^a^	(0.01)	20.89 ^a^	(5.45)	13.5 ^a^	(3.24)	0.58 ^a^	(0.12)	25.89 ^a^	(8.31)	17.06 ^a^	(4.25)	0.87 ^a^	(0.01)
**Water 100 °C**	29.82 ^b^	(3.85)	17.35 ^b^	(3.58)	0.8 ^b^	(0.03)	31.70 ^b^	(6.98)	18.02 ^b^	(2.98)	0.95 ^b^	(0.22)	41.28 ^b^	(3.02)	27.65 ^b^	(8.95)	1.87 ^b^	(0.27)
**Water 150 °C**	36.54 ^c^	(3.63)	20.67 ^c^	(3.95)	1.15 ^c^	(0.13)	39.89 ^c^	(7.23)	24.51 ^c^	(2.65)	1.38 ^c^	(0.03)	52.19 ^c^	(5.68)	35.29 ^c^	(5.64)	2.64 ^c^	(0.13)
**Water 200 °C**	45.33 ^d^	(3.90)	22.54 ^c^	(3.57)	1.65 ^d^	(0.08)	50.60 ^d^	(4.36)	31.33 ^d^	(3.58)	1.51 ^c^	(0.30)	61.34 ^d^	(4.26)	44.54 ^d^	(4.23)	3.21 ^d^	(0.53)

a–d: Different letters in the same column denote a significant difference according to the Student–Newman–Keuls test, at *p* ≤ 0.05. E:W: ethanol–water.

## Data Availability

Data is contained within the article.
